# Medical Students’ Learning About Other Professions Using an Interprofessional Virtual Patient While Remotely Connected With a Study Group: Mixed Methods Study

**DOI:** 10.2196/38599

**Published:** 2023-01-17

**Authors:** Carrie Tran, Eva Toth-Pal, Solvig Ekblad, Uno Fors, Helena Salminen

**Affiliations:** 1 Division of Family Medicine and Primary Care Department of Neurobiology, Care Sciences and Society Karolinska Institutet Stockholm Sweden; 2 Academic Primary Healthcare Centre Region Stockholm Stockholm Sweden; 3 Cultural Medicine Department of Learning, Informatics, Management and Ethics Karolinska Institutet Stockholm Sweden; 4 Department of Computer and Systems Sciences Stockholm University Stockholm Sweden

**Keywords:** interprofessional learning, virtual patient, medical students, remote learning, distance learning, medical education

## Abstract

**Background:**

Collaboration with other professions is essential in health care education to prepare students for future clinical teamwork. However, health care education still struggles to incorporate interprofessional education. Distance learning and virtual patients (VPs) may be useful additional methods to increase students’ possibilities for interprofessional learning.

**Objective:**

This study had two aims. The first was to assess if an interprofessional VP case could facilitate medical students’ learning about team collaboration in online groups. The second was to assess how students experienced learning with the VP when remotely connected with their group.

**Methods:**

A mixed methods design was used. The VP case was a 73-year-old man who needed help from different health professions in his home after a hip fracture. Questionnaires were answered by the students before and directly after each session. Qualitative group interviews were performed with each group of students directly after the VP sessions, and the interviews were analyzed using qualitative content analysis.

**Results:**

A total of 49 third-year medical students divided into 15 groups participated in the study. Each group had 2 to 5 students who worked together with the interprofessional VP without a teacher’s guidance. In the analysis of the group interviews, a single theme was identified: the interprofessional VP promoted student interaction and gave insight into team collaboration. Two categories were found: (1) the structure of the VP facilitated students’ learning and (2) students perceived the collaboration in their remotely connected groups as functioning well and being effective. The results from the questionnaires showed that the students had gained insights into the roles and competencies of other health care professions.

**Conclusions:**

This study demonstrates that an interprofessional VP enabled insights into team collaboration and increased understanding of other professions among student groups comprising only medical students. The interprofessional VP seemed to benefit students’ learning in an online, remote-learning context. Although our VP was not used as an interprofessional student activity according to the common definition of interprofessional education, the results imply that it still contributed to students’ interprofessional learning.

## Introduction

### Background

Effective interprofessional collaboration has shown a number of positive results for both patients and caregivers, such as increased patient satisfaction and the delivery of safe and patient-centered care [1,2]. Health care students’ attitudes toward interprofessional teamwork are important and are formed from experiences and interactions. Different interprofessional learning activities may influence their future practices in their chosen professions [3]. Providing training in interprofessional care is crucial for health care students to acquire the skills needed for future clinical teamwork [2,4].

There are several barriers to interprofessional education (IPE), such as geographical distance, crowded timetables, and logistical difficulties [5], and it has always been a challenge to bring students from different health professions together, especially in primary health care settings [1]. The COVID-19 pandemic created further barriers to interprofessional learning at workplaces, and social distancing has affected the learning environment at medical universities.

Distance learning is not a new phenomenon in academia [6,7], and due to the COVID-19 pandemic, transition from on-campus learning to distance learning has been required at many universities. However, there are well-known challenges with distance learning, such as technological issues and the frequent association of distance learning with decreased student engagement [8]. Nevertheless, distance learning may facilitate high-quality health education in primary health care, where students are often geographically scattered during their clinical placements [6]. Distance learning with access to online learning materials, such as virtual patients (VPs), has shown great potential in previous studies to solve logistical problems in many health education settings [9,10]. Such materials, including VPs, are convenient for students to access at any time and from any location, which might also facilitate the use of such methods. VPs are computer-based simulations of patient encounters [11] that have been successfully used globally in different settings and with different purposes in medical education [11-15].

There have been some past studies of VPs that included students from different health care professions with the aim of exploring how VPs can contribute to IPE [10,16,17]. However, there is a knowledge gap regarding team collaboration and how students of the same profession learn about other professions using VPs. Prior to COVID-19, we had already received funding for a study to investigate how an interprofessional VP could prepare medical students for real home visits. The students were intended to work with the VP in an in-person setting as part of interprofessional groups. Due to COVID-19, all health care education had to be rethought and was shifted from in-person delivery to alternatives such as remote, online learning. Unfortunately, we could not arrange interprofessional student groups on such short notice.

In this study, the aim was to investigate how a VP designed for interprofessional student groups might be useful for groups comprising solely medical students to gain insights into other professions’ competencies and into team collaboration.

### Aims of the Study

This study had two aims: (1) to assess if an interprofessional VP could facilitate medical students in learning about team collaboration in online groups and (2) to assess how students experienced learning with the VP when they were remotely connected with their group.

## Methods

### Study Design

This study was not interprofessional in the traditional sense, as we had students from only one profession participating in the study. Nevertheless, the VP used in this study was interprofessional in its design, and it had been previously studied by us in a setting with interprofessional student groups [16].

The study had a mixed methods design [18] that used both qualitative and quantitative approaches to strengthen the research findings. We performed group interviews with medical students and applied qualitative content analysis with an inductive approach to the data [19,20]. In addition, we used questionnaires to explore students’ previous knowledge of different professions and investigate how the VP contributed to their understanding of other professional roles and teamwork (Multimedia Appendix 1).

### The Interprofessional VP

The interprofessional VP case was a 73-year-old man who had recently returned home and received home care after surgery for a hip fracture. The case included 3 short illustrative video clips demonstrating individuals from 4 different health professions working together in home care with the intention to help the patient. The case also contained textual information about the roles and competencies of different health professions. During the case, students had to formulate and submit free-text answers to questions exploring their thoughts and further planning for the patient. After submission of their group reflections, the students received feedback from teachers as preprepared comments [16].

### Context and Participants

Before the COVID-19 pandemic, all students in the third year of their medical program had a compulsory assignment in which they participated in a home care visit led by a clinician from another profession. During this assignment, the students interviewed the patient, the clinician, and the home care workers. The task for the students was to identify, describe, and reflect on the roles of the professionals who participated in the care of the patient and to describe how they collaborated with each other. Due to the pandemic, the students could no longer participate in home care visits or in physical group meetings. We had trialed our VP with interprofessional student groups in physical meetings in previous years, which we described in a previous paper [16]. We decided at the beginning of the pandemic to use the same interprofessional VP in a completely new context with groups including only medical students who were remotely connected to each other. Starting from May 2020, all third-year medical students had to perform this new assignment and work with the VP case in remote group meetings as a replacement for the physical home visits.

### Recruitment

Participants in this study were recruited via the learning platform for the course. After viewing a presentation on the compulsory assignment, the students received a written invitation to participate in the study. Students who were interested could click on a link to go to the study site, which was a page on the same learning platform. On the page, the students received more detailed information and were presented with the option to sign up. Participation in the study was voluntary. Students could sign up on given dates that were indicated on a calendar, and they were encouraged to choose 2 or 3 peers with whom they wanted to work and to sign up together. They had the choice to either initiate a new group or to join an already existing group. During the VP session, the students were instructed to be at separate physical locations of their choosing and to interact exclusively via an online communication tool (Zoom; Zoom Video Communications, Inc). One student in each group, the navigator, had to open the VP system on their computer and then share the screen with their peers. Each group had to decide at the beginning of their session which of them would be the navigator. The navigator had the role of navigating the VP system according to the wishes of their peers and submitting the group’s reflections into the system. The majority of students were at home when working with the VP, while some were on campus. The students worked with the VP on their own without the presence of teachers.

### Data Collection

The sessions were limited to a maximum of 2 hours. A Zoom link was sent to each group the day before their scheduled session with the VP, and each student was asked to answer separate questionnaires before and after the VP session (Multimedia Appendix 1). The before-VP questionnaire was filled in on the learning platform. The items in this questionnaire were measured on a 6-point Likert scale, ranging from 1 (“totally disagree”) to 6 (“totally agree”). The questionnaire had demographic questions on sex, age, and prior experience of IPE activities. There were also questions about students’ prior experience of learning activities with other health professions. After the VP session, as soon as the group interview was completed, the students received an email with the after-VP questionnaire. This questionnaire contained 2 additional questions, with free-text answers in which the students could describe what they had found especially valuable about the activity and what they would have preferred to be done differently. Directly after the session, each group was interviewed via the same Zoom link used for the session. Each interview lasted from 10 to 20 minutes and used an interview guide with the following open-ended questions: “How did you perceive working with the VP model remotely?” “How did the virtual patient help you in learning about other professions?” “Was there any other profession that you would have wished to get more information about?” and “How did you perceive working with each other remotely and only one person could navigate the VP during the whole session, how did that impact your learning experience?” There was an interviewer and an observer present at each interview. A total of 11 of the 15 interviews were led by author CT while author ETP observed. The group interviews were recorded with the recording function of the Zoom app and transcribed verbatim by CT.

### Data Analysis

#### Statistical Analysis of Quantitative Data

The statistical analysis of the students’ answers to the before-VP and after-VP questionnaires was performed using Stata/BE (version 17; StataCorp LLC). Median scores with IQRs were calculated, and differences in scores before and after the learning activity were analyzed using the Wilcoxon signed rank test for paired measurements. *P* values less than .05 were considered statistically significant.

#### Qualitative Content Analysis

Qualitative content analysis was used for the analysis of the group interviews, inspired by the methods of Krippendorff [20] and Graneheim and Lundman [19]. The analysis focused on both manifest and latent content. The transcripts were initially read and reread to capture the content as a whole and were coded independently by authors CT, ETP, and HS. The material was coded manually. Meaning units relevant to the aim were identified, condensed, and labeled with codes and then discussed until consensus was reached. The various codes were interpreted and compared in a search for patterns, and codes with similar content were grouped into subcategories. The subcategories were then compared with each other and sorted into higher-level categories. The categories and subcategories can be seen as an expression of the manifest content of the text and describe the visible and obvious meanings in the text [19]. In the final stage, we tried to capture the essence of the material. A theme was formed that was considered to reflect the underlying meaning through condensed meaning units, codes, and categories on an interpretative level [19]. During the analysis process, CT, ETP, and HS moved back and forth between the whole and parts of the text. Finally, codes, subcategories, categories, and the theme were discussed until consensus was reached. Investigator triangulation was used to increase trustworthiness, and suitable quotes from different interviews were selected to illustrate the categories. In all transcriptions the students were coded, and the material was pseudonymized. Since new data did not add anything new in the last qualitative interviews, it was considered that sufficient information power was obtained for the qualitative interviews.

### Ethical Considerations

Ethical approval was obtained from the Regional Ethical Review Board in Stockholm, Sweden (Dnr 2012/1011-31/5). Due to COVID-19 constraints, the request for informed consent and the subsequent responses were communicated in writing. Students could only sign up for participation via a link from the learning platform. They received the information that signing up on the calendar for a session with the VP meant that they gave their informed consent to participate in the study. Participation in the study was voluntary, and the students were informed that participation or withdrawal from the study would not influence their future studies or contact with the university. ETP had contact with the students both as a teacher and an interviewer. HS was responsible for the primary care component of the study program in medicine. Ethical issues related to the double roles of ETP and HS were discussed, and the group interviews were mostly performed by CT.

## Results

### Quantitative Results

A total of 244 students were eligible for the study, 49 of whom signed up to participate, including 18 men and 31 women. They formed 15 groups with 2 to 5 students in each. The median age of the participants was 25 (range 20-50) years. Of the 49 students, 46 answered the question about whether they had had experience of learning activities together with other professions, and 31 of these 46 (67%) had no previous interprofessional experience. There were 15 students who reported having had such experiences with nursing students. The results from the before-VP and after-VP questionnaires are presented in Table 1. The students reported having increased their understanding of the roles of the other professions presented in the VP case. The largest increase was in the perceived understanding of the role and competence of occupational therapists.

**Table 1:** The answers of students (N=49) to questionnaire items before and after working with an interprofessional virtual patient. Items were scored on a 6-point Likert scale, ranging from 1, (“totally disagree”) to 6, (“totally agree”).

Items				Before VP^a^ session score, median (IQR)		After VP session score, median (IQR)		*P* value^b^
**Items related to insight into the roles of various professions^c^**								
		Family physician	3 (2-4)		5 (5-6)^d^		*<*.001	
		District nurse	3 (2-4)		5 (5-6)^d^		*<*.001	
		Physiotherapist	3 (1-4)		5 (5-6)^e^		*<*.001	
		Occupational therapist	2 (1-4)		6 (5-6)^d^		*<*.001	
		Community-based home care	4 (4-6)		4 (4-5)^d^		.68	
**Items related to insight into the collaboration between professionals^f^**								
	District nurse and family physician		2 (2-3)		5 (4-6)^d^		*<*.001	
	Physiotherapist and occupational therapist		1 (1-2)		6 (5-6)^d^		*<*.001	
	Community-based home care and health care professions		2 (2-3)^g^		4 (3-5)^d^		*<*.001	
**Other items**								
	“I have good information technology skills”		5 (4-5.5)^g^		N/A^h^		N/A	
	“I am familiar with using VPs and simulations for learning”		3 (2-4)^g^		N/A		N/A	
	“Working together with the VP remotely functioned well”		N/A		6 (5-6)^d^		N/A	
	“Our discussions contributed to my learning about the different professions’ roles in a home visit”		N/A		5 (5-6)^d^		N/A	
	“I perceive that working with the VP helped me to be better prepared for a real home visit”		N/A		5 (4-6)^e^		N/A	

^a^VP: virtual patient.

^b^Determined with the Wilcoxon signed rank test for paired measurements.

^c^Item before the session: “I perceive that I have insight into the role of the following professions during a home care visit”; item after the session: “I perceive that I have a more in-depth insight into the role of the following professions during a home care visit.”

^d^Data missing from 2 students.

^e^Data missing from 3 students.

^f^Item before the session: “I perceive that I have insight into the collaboration between...”; question after the session: “I perceive that I have a more in-depth insight into the collaboration between...”

^g^Data missing from 1 student.

^h^N/A: not applicable. This question was not asked in this session.

### Qualitative Results

From the analysis of the group interviews, we identified a single theme: the interprofessional VP promoted student interaction and gave insight into team collaboration. Two categories were found: (1) the structure of the VP facilitated students’ learning and (2) students perceived the collaboration in their remotely connected group as functioning well and being effective (Textbox 1).

**One theme, 2 categories, and 9 subcategories were identified from the 15 group interviews with medical students.**

**Theme: The interprofessional virtual patient promoted student interaction and gave insight into team collaboration.**

For the category “the structure of the virtual patient facilitated students’ learning,” subcategories included the following:

- A mix of different methods with the virtual patient promotes learning.
- The virtual patient provides an understanding of the students’ own and other professionals’ roles and responsibilities.
- The virtual patient provides insights into the importance of collaboration between professions.
- The virtual patient provides as much information about handling the patient case as a real home visit.

For the category “students perceived the collaboration in their remotely connected group as well-functioning and effective,” subcategories included the following:

- Work with the virtual patient remotely was effective.
- Roles were distributed.
- It was good for the students to be able to choose who they wanted to work with.
- The students’ experiences of communication in the group during the session.
- The discussion would have been richer if there were other professions in the group.

### The Interprofessional VP Promoted Student Interaction and Gave Insight Into Team Collaboration

This theme described how the interprofessional VP generated interactions between the students in several ways. The students felt that they could get help from each other by sharing their previous experiences and knowledge about other professions and in this way be able to help the patient in the VP exercise. The students had to formulate their thoughts about the patient’s situation and how they would help the patient and then had to submit their reflections in the system, which prompted them to discuss their thoughts and reflections within the group. The students reported that the mixture of video clips, text, and free-text responses in the structure of the VP prompted them to discuss and interact with each other regularly during the session. The students perceived that the VP case gave them insights into team collaboration by providing them with information about how different professions acted and collaborated in the case (via the video clips) and about those professions’ competencies in general (via the texts).

### The Structure of the VP Facilitated Students’ Learning

All of the students appreciated the short video clips in the VP case because they felt that the clips helped them to obtain a detailed understanding of how other professions act in their roles during home visits. The students found the VP case to be realistic and that it gave a sense of meeting a real human being. The students also appreciated the texts in the VP that contained information about different professions’ competencies, because these texts complemented the videos. The students reported that it became clear to them from the case how different health professions contributed to helping the patient in an optimal way. This made the students understand the importance of collaboration:

How much one succeeds when there are several professionals working together depends on maybe several factors such as how you collaborate, how you listen to each other, or how you can complement someone if someone has forgotten something.

Student group 15

The students perceived having learned more about other professions by actively discussing the VP case in the group than if they had been listening to a lecture:

If you compare this way and think about all the professions compared to maybe sitting in a lecture where they talk about what different professions do during a home visit, then you would have zoned out immediately. But now you look and sort of discuss, laugh, and think as well. I think you learn a lot more this way.

Student group 10

The students reported that they had gained insights into other professions’ roles and responsibilities by working with the VP. Several of the students mentioned explicitly that they had learned the differences between the roles of physiotherapists and occupational therapists. They also mentioned that it was new information for them that doctors can examine patients in their homes. The students found it valuable that the VP case provided them with information about professions that they would not have received if they had participated in a real-life home visit. Working with the VP made them feel more active in the patient consultation compared to a real home visit, during which they would typically listen passively to their supervisor:

This is an easier way to stay active throughout the task, and therefore to get more out of it than if you just sit passively next to someone and listen.

Student group 2

### Students Perceived the Collaboration in Their Remotely Connected Group as Functioning Well and Being Effective

In this category, the students described how using the VP remotely functioned well. They almost perceived being in the same room together, because all of the participants in the group could follow the VP on their own screen:

I do not think there would be any major difference if we had been sitting together. This way we could all sit and watch the screen and follow along.

Student group 11

Although only one of them acted as navigator and ran the VP, the students found it easy to work together. Indeed, some of them thought that it would lead to problems with teamwork if all of them could run the VP simultaneously. The students who navigated the VP needed to be responsive to their peers in the group discussions:

We discussed together what we should write and answer, so it was “B” who wrote everything, but we also said, like, “Ah, but you can write this and do it like this”...and so we always checked with each other by asking, “Are we ready click on the next section?”

Student group 14

Those who did not run the VP felt that they were still actively involved in the group discussions. A spontaneous comment from some students was that they appreciated the opportunity to choose which peers they worked together with:

We could choose who we would be with, which also contributed to us...we all three know each other so we know a little about our dynamics, our prior knowledge, and that may be why it went so fast for us, too...ehh, because it will be a lot more efficient when you already know about the others, we have worked with them a bit before.

Student group 2

However, the students stated that they would have appreciated working on the VP together with students from other professions. They wanted to know how students from other professions would have reasoned about the issues being discussed. In the absence of other professions, the students had to try to imagine the other professions’ perspectives.

The students reported that their experience of working remotely was mainly positive, and they reported feeling more relaxed and saving travel time. However, the students also mentioned some difficulties working remotely, such as not being able to notice on the screen when someone wanted to speak. They also mentioned the importance of technology that functioned well, although they rarely experienced any technical problems.

A total of 44 students gave free-text responses about what they thought was especially valuable about the learning activity. The answers were in accord with the findings from the group interviews. They described how the short video clips helped them to better understand the roles and competencies of other health professions, and they also stated that they would like to work on the VP case with students from the other professions that were presented in the VP.

## Discussion

### Principal Results

To our knowledge, this is the first study that has assessed how an interprofessional VP can contribute to medical students’ learning about other professions and about team collaboration while remotely connected in online groups. The students perceived that the VP promoted learning and interaction in their groups and gave insight into team collaboration. The mixture of visual and textual information in the VP added valuable knowledge about the involved professions and was highly appreciated by the students. The students found that the VP functioned well and that it was effective to work with the VP while remotely connected to their groups. They stated that the digital communication tool allowed their conversations to flow smoothly, because they could see and hear each other on the screen. The students appreciated the video clips that demonstrated how different health professions collaborated with each other in home care, and they expressed the opinion that the video clips added greatly to their understanding of the roles and competencies of other professions.

### Comparison With Prior Work

The finding that embedded video clips could facilitate students’ interprofessional learning is in accord with our previous study [16], in which students worked with the same interprofessional VP case in face-to-face interprofessional student groups. Students in both studies reported similar perceptions about how the VP facilitated their interprofessional learning. The methods described in this study may not match the traditional notion of IPE because the student groups comprised only medical students, but our findings show that they still gained insights into team collaboration and a better understanding about other professions’ competencies.

In a study by Edelbring et al [21], students could choose either to have a real-life meeting or to meet online in their interprofessional learning activity with a VP. The majority of the students chose to meet online and expressed the feeling that it worked well to have a digital meeting. The finding that the students appreciated working online is in line with our findings in this study.

Most published studies of IPE have involved at least two different health professions and have reported mostly positive findings, such as students stating that collaboration across professions benefits patients and helps to clarify professional roles [22-25]. We obtained similar results in our study even though we only had students from one profession. The VP case thus supported the students in understanding the importance of team collaboration to help patients.

Some students appreciated the opportunity to choose the peers they wanted to work with, and they found the discussions to be easier and more rewarding when working with peers they knew. Whether or not it is beneficial to be able to choose your working partners could, however, be questioned, and in their upcoming professional roles, new clinicians should be able to collaborate with people they are not familiar with. Our students appreciated being able to discuss the care of the patient with their peers and obtain immediate feedback from the VP system. In studies by Croft et al [26] and Dost et al [27], students reported barriers to online learning, such as not having peers for discussion and lacking immediate feedback from a teacher. The interprofessional VP described in this study cannot replace traditional IPE, but it could meet the challenges faced by every faculty, such as logistical difficulties and the recent challenges of social distancing due to COVID-19. After this study was completed, the interprofessional VP was implemented as a permanent learning activity for all third-year medical students.

### Limitations

A limitation was that the questionnaire used before and after the learning activity was not validated or pilot tested prior to its use, due to a lack of time at the start of the study. Another potential limitation is that two of the researchers were involved in the medical program as teachers; hence, the teacher-student relationship might have influenced the study participants’ questionnaire answers. Additionally, the students were able to choose their peers for the groups by themselves, rather than being randomly assigned, and this could also be seen as a limitation. Furthermore, because the students in this study participated on a voluntary basis, it is not known how nonparticipating students would have perceived working with the VP online remotely in groups. On the other hand, 49 of the 244 eligible students participated in the study, and the findings may have been similar among students who did not participate, because they performed exactly the same activity under the same circumstances and expressed a high level of appreciation in their course evaluation. In other words, the study was performed in a real-life context. Forty-nine of 244 eligible medical students may be considered a small sample size in quantitative research, but the sample size was large for a qualitative study. This study was also limited by the participation of medical students from a single university; therefore, the results might not be transferrable to medical students from other universities.

### Conclusions

The results of this study demonstrate that the interprofessional VP gave insight into team collaboration and increased the understanding of other professions among student groups comprised only of medical students. The interprofessional VP seemed to benefit students’ learning in an online, remote-learning context. Though our VP was not used as an interprofessional student activity according to the definition of IPE, the results imply that it still contributed to students’ interprofessional learning.

## Appendix

Multimedia Appendix 1 Questions to the students in the individual questionnaire before and after working with the interprofessional virtual patient.

## Abbreviations

IPE: interprofessional education
VP: virtual patient
