# Supplementary material for: Medical Students’ Learning About Other Professions Using an Interprofessional Virtual Patient While Remotely Connected With a Study Group: Mixed Methods Study
Source: JMIR Med Educ. 2023 Jan 17;9:e38599. doi: 10.2196/38599 (PMC9890351; doi:10.2196/38599)
Supplement: Multimedia Appendix 1 [file mededu_v9i1e38599_app1.docx]

**Multimedia Appendix 1: Questions to the students in the individual questionnaire before and after working with the interprofessional virtual patient.**

| questions before | | questions after |
| --- | --- | --- |
| Q1 | I have insight into the role of a family physician in a home visit | I have deepened my understanding of the role of a family physician in a home visit |
| Q2 | I have insight into the role of a district nurse in a home visit | I have deepened my understanding of the role of a district nurse in a home visit |
| Q3 | I have insight into the role of a physiotherapist in a home visit | I have deepened my understanding of the role of a physiotherapist in a home visit |
| Q4 | I have insight into the role of an occupational therapist in a home visit | I have deepened my understanding of the role of an occupational therapist in a home visit |
| Q5 | I have insight into community-based home care | I have deepened my understanding of the role of community-based home care |
| Q6 | I have insight into how doctors and district nurses collaborate in a home visit | I have deepened my understanding of how family physicians and district nurses collaborate in a home visit |
| Q7 | I have insight into how physiotherapists and occupational therapists collaborate in a home visit | I have deepened my understanding of how physiotherapists and occupational therapists collaborate in a home visit |
| Q8 | I have insight into what collaboration looks like between community-based home care and the health care professions | I have deepened my understanding of what collaboration looks like between community-based home care and the health care professions |
| Q9 | I have good IT skills | Working together with the VP remotely functioned well |
| Q10 | I am used to using VPs/simulations for learning | Our discussions during the session contributed to my knowledge about the role of other professions in a home visit |
| Q11 |  | Working with the VP has helped prepare me for a real home visit |
| Q12 |  | Was there anything you found especially good with this learning activity? ^*^ |
| Q13 |  | Was there anything in this learning activity you would have preferred to be done differently? ^*^ |

^*^ Questions with free text answers
